# Higher Genetic Diversity Across Several Small Patches Compared to a Single Large Patch: A Within‐Species Test of SLOSS

**DOI:** 10.1111/eva.70229

**Published:** 2026-03-24

**Authors:** Fumin Lei, Dandan Li, Hua Zhu, Yuzhu Xia, Xinyi Chen, Jianxiang Yu, Shuiliang Guo, Jing Yu

**Affiliations:** ^1^ College of Life Sciences Shanghai Normal University Shanghai People's Republic of China; ^2^ Yangtze River Delta Urban Wetland Ecosystem National Field Scientific Observation and Research Station Shanghai Normal University Shanghai People's Republic of China

**Keywords:** bryophyte, genetic diversity, haplotype, phylogenetic diversity, SLOSS, Zhoushan archipelago

## Abstract

The SLOSS (Single Large or Several Small) debate is crucial in reserve design, focusing on evaluating the conservation efficacy of a single large reserve versus multiple small reserves for species preservation. While accumulating evidence suggests that multiple small reserves may conserve greater species diversity, the question of how to maximize the conservation of intraspecific genetic diversity within a fixed total area remains largely unexplored. This study evaluated the SLOSS strategy for conserving intraspecific genetic diversity in *Tortula muralis* using haplotype richness and phylogenetic diversity metrics. We collected 352 specimens from 28 islands within the Zhoushan Archipelago, China. Amplification of the ITS2 region yielded 78 distinct haplotypes. Following the construction of a haplotype‐based phylogenetic tree, phylogenetic diversity was calculated for each island. Cumulative curves for both haplotypes and phylogenetic diversity were generated by sequentially adding islands ranked by area, following both largest to smallest and smallest to largest sequences. The results demonstrate that the genetic diversity of *T. muralis* is influenced by both island area and anthropogenic activities. Crucially, within a fixed total area, multiple small islands preserved significantly greater haplotype richness and phylogenetic diversity of *T. muralis* compared to a single large island (SS > SL). This study provides valuable insights for assessing the SLOSS strategy to conserve intraspecific genetic diversity and offers a new perspective for genetic diversity conservation.

## 1 Introduction

The design of protected areas is a complex process, with its theoretical origins traceable to early guidelines based on simple ecological principles, the universal applicability of which has been challenged by numerous studies (Fahrig et al. 2022). Although conventional wisdom, particularly inferences drawn from island biogeography theory and the species‐area relationship, often advocates for maximizing the size of protected areas, and area is generally considered a key predictor of species richness (MacArthur and Wilson 1967; Diamond 1976; Tjørve 2010; La Sorte et al. 2020), expanding protected area is thus usually beneficial for accommodating more species (Fahrig et al. 2022; Riva and Fahrig 2023). However, empirical evidence indicates that this is not always the case. Collections of multiple small patches may surpass single large patches in terms of species richness, although not necessarily in phylogenetic or functional diversity (Fahrig et al. 2022; Rösch et al. 2015). The ongoing debate over whether a Single Large (SL) area or Several Small (SS) areas more effectively support species diversity within a fixed total area is commonly referred to as the SLOSS (Single Large or Several Small) debate.

Most studies have focused on how to maintain higher species richness within limited areas, finding that the SS > SL configuration represents a predominant pattern across diverse taxonomic groups and geographical regions (Deane 2022; Fahrig 2003; Fahrig et al. 2022). When conservation aims specifically target the preservation of individual endangered species, a key question arises: for such a species, how can we maximize the preservation of genetic diversity within a fixed area? Previous studies have demonstrated significant positive correlations between population‐level genetic diversity and habitat patch size (Dixo et al. 2009; Fan et al. 2024). However, these studies have primarily focused on the effects of single‐patch area or fragmentation per se on genetic parameters, leaving unexplored their relative efficiency in conserving intraspecific genetic variation explicitly within the SLOSS conservation framework.

Theoretically, island area sets a baseline for genetic diversity by modulating effective population size, with larger islands generally expected to maintain higher diversity (Costanzi and Steifetten 2019; Fan et al. 2024). However, the spatial configuration of habitat patches can strongly modify this area effect by influencing key ecological and evolutionary processes. First, the degree of patch isolation affects the sources and frequency of colonization; stochastic colonization events from divergent source populations can generate heterogeneous founder effects across islands, implanting unique alleles at fine spatial scales (Waters et al. 2013). Second, the size and distribution of patches directly govern population dynamics. Compared to large islands, populations on small islands are smaller, more susceptible to genetic drift, and undergo more frequent extinction‐recolonization cycles (MacArthur and Wilson 1967). This dynamic turns small‐island populations into both “filters” and “reservoirs” of genetic variation—they may rapidly lose some variants due to drift and founder effects but may also stochastically accumulate a rich pool of rare alleles through repeated recolonizations from diverse sources. For instance, the geographic isolation of unique haplotypes in the lesser rice‐field rat across small islands documented by Liu et al. (2019) exemplifies this spatially driven process of genetic differentiation. Therefore, synthesizing these evolutionary processes governed by landscape configuration, we aim to test the following core hypothesis within the SLOSS framework: under a fixed total area, the assemblage of populations across several small islands (SS) will sustain greater total genetic diversity than the population on a single large island (SL) of equivalent area.

As a basal lineage of land plants (McDaniel 2021), bryophytes exhibit distinctive diversity distribution patterns shaped collectively by their non‐vascular nature, unique physiological‐ecological traits, and haploid‐dominant life cycle strategy. Compared to vascular plants, the gametophyte‐dominant life history of bryophytes means that their genetic mutations are directly exposed to natural selection during the haploid phase, thereby increasing the efficiency of selection (Gerstein and Otto 2009; Yuan et al. 2025). This increased efficiency allows for the rapid elimination of deleterious mutations or fixation of beneficial variants, consequently shaping the genetic diversity patterns of populations (Lueth and Reski 2023; Yuan et al. 2025).

To test the hypothesis that, under an equivalent total area, Several Small (SS) islands preserve greater intraspecific genetic diversity than a Single Large (SL) island, we selected *Tortula muralis* Hedw. in continental islands as the study system. First, prior research has documented the widespread distribution of *T. muralis* across numerous islands in the Zhoushan Archipelago (Yu et al. 2019), providing the requisite replicated population samples across an island size gradient needed to test SLOSS configurations. Second, as a typical wall‐dwelling bryophyte, *T. muralis* predominantly colonizes concrete walls and similar anthropogenic structures across the archipelago (Gao 1996). This reliance on a widespread, functionally similar substrate across islands minimizes the confounding effect of major natural habitat heterogeneity. Consequently, observed genetic diversity patterns are more likely attributable to island area and spatial configuration, which are the core variables of the SLOSS framework, rather than to stark differences in habitat type. This makes it an ideal model for isolating and testing the effects of reserve size and arrangement themselves. Specifically, this study aims to: (1) quantify and compare the haplotype richness and phylogenetic diversity of *T. muralis* populations under simulated SL versus SS configurations within the Zhoushan Archipelago; and (2) evaluate the influence of island environmental factors on the distribution of genetic diversity to contextualize the SLOSS comparison.

## 2 Material and Methods

### 2.1 Specimen Collection

The Zhoushan Archipelago, the largest archipelago in China, is situated in the northeastern part of Zhejiang Province (29°32′–31°04′ N, 121°30′–123°25′ E). This paper is one in a series of studies investigating the bryophyte flora and biogeography of this archipelago. The general background of the study area has been described in preceding papers (Yu et al. 2019, 2020).

**FIGURE 1:**
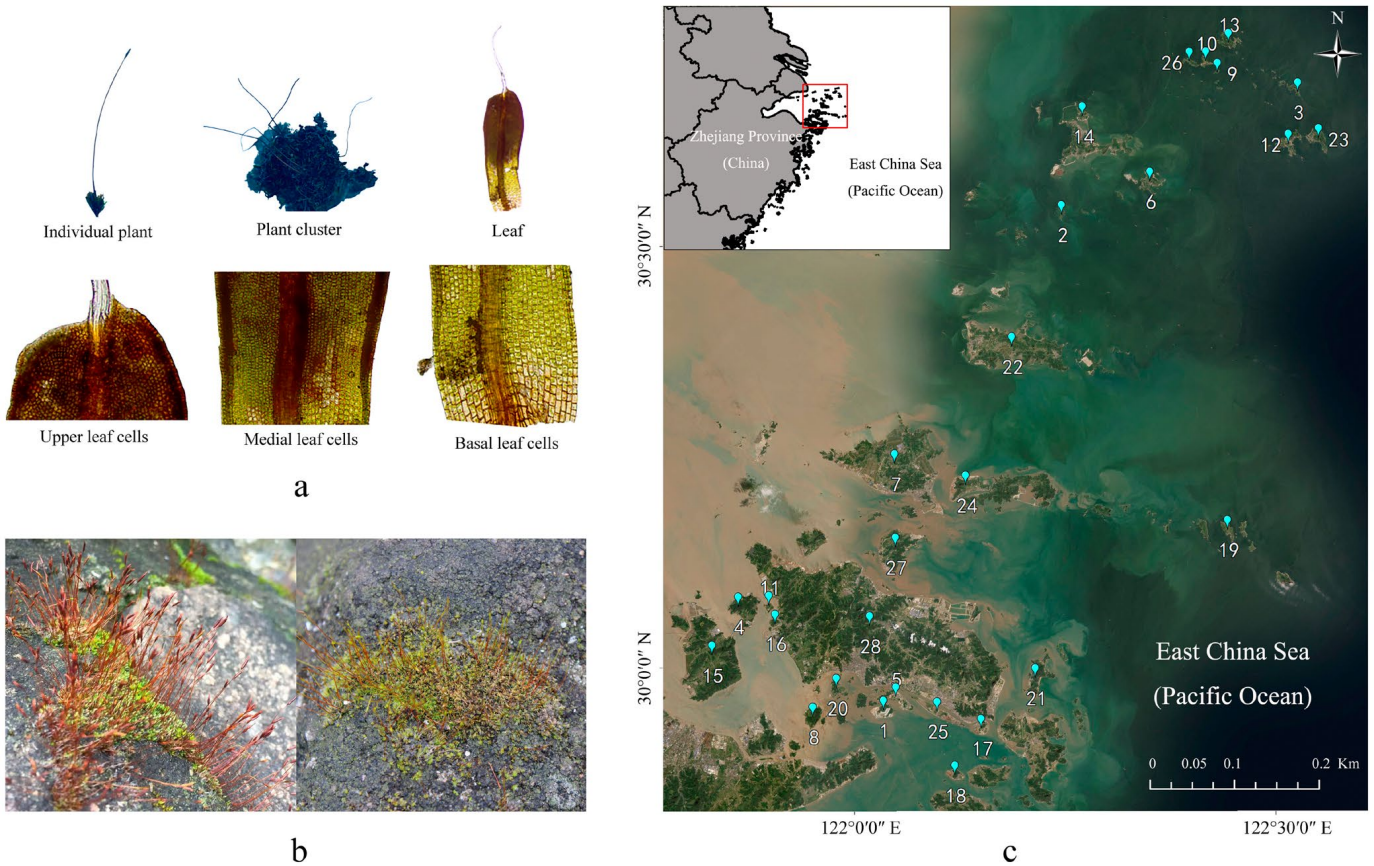
The illustration of *T. muralis* (a), the sampling habitat (b), and the locations of the 28 sampling islands (c).

We selected *T. muralis* (Pottiaceae) as our model species. This species has a cosmopolitan distribution pattern and is commonly found on calcareous rocks and artificial concrete walls, making it a characteristic “wall‐dwelling” bryophyte. In the field, *T. muralis* is readily identifiable by its diminutive stature and the elongated, hyaline hair‐points formed by excurrent costae (Gao 1996) (Figure 1a,b).

To sample *T. muralis* populations, we conducted five field campaigns across the Zhoushan Archipelago between 2014 and 2021 (specifically in May 2014, July–August 2016, July–August 2017, and October 2021). Based on preliminary surveys, we stratified sampling across three occupied habitat types: rock surfaces, north‐facing slopes, and anthropogenic structures. To ensure sample independence and minimize clonal sampling, specimens within the same habitat category were collected at a minimum spacing of 50 m. Each specimen was placed and stored in a separate, new paper specimen bag immediately upon collection. Specimen collection encompassed 28 islands with a total of 352 samples, where sampling intensity increased proportionally with island size to account for greater habitat heterogeneity. All collected materials were air‐dried in the laboratory and subsequently re‐examined under microscopy for taxonomic verification. Voucher specimens are deposited at the Bryophyte Herbarium, College of Life Sciences, Shanghai Normal University (SHTU).

### 2.2 Molecular Amplification

The internal transcribed spacer 2 (ITS2) region spanning the 5.8S‐26S ribosomal RNA genes was amplified, consistent with its established utility in bryophyte phylogenetic studies (Leonardía et al. 2013; Merget and Wolf 2010). Total genomic DNA was extracted directly from individual *T. muralis* gametophytes using the Thermo Scientific Phire Plant Direct PCR Kit (Thermo Fisher Scientific), followed by PCR amplification according to manufacturer specifications. Amplification products were purified and sequenced unidirectionally by BGI (Beijing Genomics Institute; www.genomics.cn) using the original PCR primers. The following primers were employed: 5.8SF: 5′‐GACTCTCAGCAACGGATA‐3′; 26SR: 5′‐AGATTTTCAAGCTGGGCT‐3′.

Previous studies on *T. muralis* and congeners have documented the occurrence of ITS pseudogenes (Košnar et al. 2012). Consistent with these reports, chromatograms from approximately one‐third of our ITS2 amplifications exhibited overlapping peaks, indicative of potential heterozygosity or multiple sequence variants. For chromatograms displaying bimodal peak patterns, triplicate independent PCR amplifications and sequencing reactions were performed. The consensus sequence derived from the dominant peaks reproducible in at least two technical replicates was adopted as the representative sequence for each specimen.

Chromatograms were analyzed using the R package SangerseqR (Hill et al. 2014), which performs base calling by evaluating the secondary‐to‐primary peak signal ratio. Specifically, for non‐repetitive regions, sequences were designated as potential heterozygous genotypes when secondary peaks exceeded 50% of the primary peak height. For repetitive regions, this threshold was reduced to 30%. These criteria align with the default parameters of SangerseqR's makeBaseCalls() function, which distinguishes significant secondary peaks from background noise using a 0.33 ratio threshold (33%). Following base calling, dominant peak sequences were extracted and aligned using MEGA X (Kumar et al. 2018) and PhyDE (Müller and Quandt 2010).

### 2.3 Data Analysis

Aligned sequences were analyzed in DNAsp v5.10 (Librado and Rozas 2009) to extract three key genetic diversity metrics: (i) haplotype number (H), (ii) haplotype diversity (Hd), and (iii) nucleotide polymorphism (Pi). Additionally, pairwise nucleotide substitutions between distinct haplotypes were systematically computed.

Haplotype sequences were used to reconstruct phylogenetic relationships under Bayesian inference in MrBayes 3.2.6 (Ronquist et al. 2012). The optimal nucleotide substitution model, selected by the Akaike Information Criterion (AIC) in MrModeltest 2.4, was “GTR + I + G”. Markov Chain Monte Carlo (MCMC) analyses comprised two independent runs, each with four chains, for 1,000,000 generations sampled every 100 generations. Phylogenetic diversity indices for *T. muralis* populations on individual islands were quantified by summing the branch lengths connecting all constituent haplotypes within the consensus tree, implemented via the R package *picante* (Kembel et al. 2010).

To investigate the influence of island characteristics on genetic diversity, we initially compiled nine environmental variables: island area (AREA), coastline length (COA), shape index (SHA), vegetation coverage (VEG), rock coverage (ROCK), distance to the nearest island (ISD), land‐to‐water area ratio (ISW), human population size (POP), and transportation frequency level (FRE). The environmental variables were sourced from Yu et al. (2019, 2020) (AREA, SHA, VEG, ROCK, ISD, and ISW) and the The Editorial Board of the Island Chronicles of China (2014a, 2014b) (COA, POP, and FRE). Collinearity among these variables was assessed using permutation‐based variance inflation factor (VIF) diagnostics with 999 iterations, leading to the exclusion of three variables (SHA, VEG, and ROCK) that exhibited high multicollinearity (VIF ≥ 10) (Liu et al. 2022). Variables that passed this diagnostic (VIF < 10) were considered nonredundant. The remaining six nonredundant environmental variables (AREA, COA, ISD, ISW, POP, and FRE) were retained for all subsequent analyses (Table 1).

**TABLE 1:** Environmental factors incorporated in this study for the island system.

ID	Island	LON	LAT	AREA	COA	ISD	ISW	POP	FRE
1	Aoshan	122.15	29.96	4.90	11.50	4.55	0.09	1087	4
2	Baijieshan	122.42	30.62	0.54	4.90	52.21	0.63	2	2
3	Bixiashan	122.78	30.79	1.18	8.61	77.15	0.97	162	3
4	Cezi	121.93	30.09	14.10	23.20	12.62	0.22	6334	5
5	Changzhi	122.17	29.97	6.10	14.00	7.40	0.08	4419	5
6	Dahuanglong	122.57	30.67	5.32	21.09	60.98	0.75	6910	5
7	Daishan	122.16	30.28	106.29	87.17	45.24	0.23	111,765	5
8	Damao	122.04	29.95	6.20	12.40	4.82	0.15	455	3
9	Dongkushan	122.66	30.81	0.23	2.88	66.86	0.92	202	3
10	Donglvhua	122.65	30.83	1.12	8.07	64.54	0.93	227	2
11	Fuchi	121.97	30.1	1.10	5.80	14.70	0.19	602	3
12	Gouqi	122.74	30.73	5.85	21.92	77.32	0.90	7611	5
13	Huaniao	122.68	30.85	3.62	16.82	67.55	0.96	977	4
14	Jinjishan	122.46	30.76	1.99	8.82	48.39	0.78	2601	5
15	Jintang	121.88	30.02	77.30	48.70	3.33	0.27	42,012	5
16	Lidiao	121.98	30.07	1.60	5.60	16.80	0.39	216	3
17	Lujiazhi	122.3	29.94	3.20	7.28	15.00	0.56	7938	4
18	Mayi	122.26	29.87	2.10	8.70	14.16	0.211	5814	5
19	Mazhi	122.24	29.95	4.20	14.15	11.06	0.51	294	3
20	Miaozihu	122.68	30.19	2.60	10.80	62.79	0.55	806	4
21	Panzhi	122.07	29.99	3.80	9.70	8.61	0.10	2045	5
22	Putuoshan	122.38	30.00	11.70	32.70	25.09	0.24	4823	5
23	Qushan	122.35	30.44	62.85	90.45	59.03	0.44	53,016	5
24	Shengshan	122.82	30.72	4.20	18.58	82.79	0.95	8309	5
25	Xiaochangtu	122.28	30.26	13.18	19.38	40.00	0.56	19,750	5
26	Xilvhua	122.63	30.82	1.28	8.92	61.41	0.91	336	4
27	Xiushan	122.16	30.17	22.66	36.96	27.24	0.12	10,106	5
28	Zhoushan	122.15	30.05	490.40	155.50	10.44	0.00	635,595	5

*Note:* Longitude (LON) and latitude (LAT) represent the geographic coordinates of each island. Island area (AREA, km^2^) denotes the total land surface extent. Coastline length (COA, km) quantifies the perimeter of the island. Interisland distance (ISD, km) indicates the distance to the nearest neighboring island. Land‐to‐water area ratio (ISW) represents the proportion of terrestrial to aquatic area within a 1‐km radius buffer centered on the island's geometric centroid. Human population size (POP) refers to the number of permanent residents. Transportation frequency level (FRE) serves as a categorical measure of accessibility intensity.

To quantify the relative contributions of island characteristics to both the composition and distribution of *T. muralis* haplotypes, we conducted hierarchical partitioning based on RDA using the rdacca.hp. package (Lai et al. 2022) to identify the independent contributions of each environmental factor. Mantel tests quantifying associations between island environmental variables and genetic diversity indices (Haplotype richness, Haplotype diversity, Nucleotide polymorphism, Phylogenetic diversity) were implemented using the *dplyr* package (Wickham et al. 2023). General linear models (GLMs) were subsequently fitted to visualize significant relationships. For all statistical tests conducted in this study, statistical significance was evaluated at the 0.05 level.

Following the protocol of Quinn and Harrison (1988), we constructed diversity accumulation curves ordered from the largest to the smallest (LTS) and from the smallest to the largest (STL) island area to characterize the accumulation patterns of haplotype richness and phylogenetic diversity under different protected area design scenarios. These curves simulate the genetic diversity preserved when prioritizing the conservation of a single large area (LTS) versus multiple small areas (STL) within a fixed total area. If the STL curve consistently remains above the LTS, it indicates that the SS strategy holds a greater advantage.

Subsequently, the SLOSS configuration was evaluated by using the discriminant metric *ζ* (Mac Nally and Lake 1999) to compare the areas under these curves, which quantifies the magnitude of the difference:

ζ=Ψ/ΔA

where *Ψ* represents the signed integral of the area difference between the STL and LTS curves, and *ΔA* denotes the total spatial extent over which the areas are computed. A positive *ζ* value indicates that, per unit area, the STL (SS) strategy accumulates diversity more efficiently than the LTS (SL) strategy, thus supporting the hypothesis that several small reserves are superior. Conversely, a negative *ζ* value would support the superiority of a single large reserve. The magnitude of *ζ* quantifies the net deviation in diversity accumulation efficiency between the two strategies.

For intraspecific diversity, STL and LTS curves closely resemble species‐area accumulation patterns in configuration. Since phylogenetic diversity was calculated independently for each island population and cannot be summed directly, haplotype counts were first accumulated following both ascending and descending orders of cumulative island area. Phylogenetic diversity indices were subsequently computed at each accumulation node using the consensus phylogeny. All analyses were implemented in R 4.2.1(R Core Team 2022), with visualizations generated using the ggplot2 package (Wickham 2016).

## 3 Results

A total of 78 haplotypes were identified across all specimens. Pairwise nucleotide substitution rates ranged from 0.002 to 0.34 (Table 2). Island‐endemic haplotypes accounted for 87% (68/78) of the total diversity. After excluding the largest island (Zhoushan Island), small islands harbored 57 endemic haplotypes, representing 73.07% of all haplotypes. Zhoushan Island contained 15 haplotypes, with the dominant haplotype H01 representing 75.41% of its specimens (Supporting Information). Phylogenetic diversity metrics for *T. muralis* populations on each island were derived from the consensus phylogeny and haplotype occurrence matrix (Table 3).

**TABLE 2:** Maximum (MAX) and minimum (MIN) nucleotide substitution rates detected among the 78 haplotypes based on ITS2 sequence variation.

Haplotype	Max	Min	Haplotype	Max	Min	Haplotype	Max	Min
H01	0.239	0.002	H27	0.248	0.009	H57	0.236	0.002
H02	0.257	0.059	H28	0.241	0.002	H58	0.336	0.173
H03	0.243	0.005	H29	0.225	0.002	H59	0.259	0.023
H04	0.243	0.002	H31	0.313	0.032	H60	0.239	0.020
H05	0.227	0.002	H32	0.221	0.009	H61	0.234	0.029
H06	0.340	0.101	H33	0.243	0.002	H62	0.232	0.002
H07	0.302	0.095	H34	0.223	0.007	H63	0.279	0.065
H08	0.227	0.002	H35	0.243	0.002	H64	0.329	0.097
H09	0.309	0.097	H36	0.227	0.029	H65	0.252	0.018
H10	0.225	0.009	H37	0.248	0.018	H67	0.225	0.007
H11	0.225	0.002	H38	0.259	0.018	H69	0.259	0.025
H12	0.241	0.002	H40	0.223	0.002	H70	0.340	0.115
H13	0.230	0.002	H41	0.248	0.025	H73	0.232	0.025
H14	0.234	0.002	H42	0.243	0.005	H75	0.241	0.002
H15	0.239	0.002	H43	0.225	0.005	H77	0.243	0.002
H16	0.241	0.002	H44	0.234	0.016	H78	0.232	0.002
H17	0.259	0.047	H45	0.234	0.018	H79	0.234	0.007
H18	0.230	0.002	H46	0.232	0.002	H80	0.227	0.023
H19	0.230	0.005	H47	0.232	0.002	H81	0.245	0.014
H20	0.241	0.002	H48	0.297	0.032	H82	0.309	0.072
H21	0.232	0.005	H49	0.239	0.002	H83	0.225	0.005
H22	0.268	0.034	H50	0.282	0.092	H86	0.234	0.011
H23	0.257	0.074	H53	0.239	0.002	H87	0.255	0.043
H24	0.239	0.007	H54	0.232	0.007	H88	0.239	0.002
H25	0.234	0.020	H55	0.227	0.007	H89	0.252	0.045
H26	0.230	0.023	H56	0.232	0.002	H90	0.241	0.002

**TABLE 3:** Specimen number (SN), haplotype richness (*H*), haplotype diversity (*H*_d_), nucleotide polymorphism (*P*_i_), and phylogenetic diversity (PD) for *T. muralis* across 28 islands.

ID	Island	Sn	*H*	*H*_d_	*P*_i_	PD
1	Aoshan	14	1	—	0.000	0.001
2	Baijieshan	1	1	—	0.000	0.001
3	Bixiashan	4	1	—	0.000	0.001
4	Cezi	19	6	0.468	0.012	0.111
5	Changzhi	17	2	0.118	0.002	0.015
6	Dahuanglong	15	10	0.857	0.032	0.172
7	Daishan	24	15	0.837	0.050	0.589
8	Damao	17	2	0.118	0.005	0.036
9	Dongkushan	1	1	—	0.000	0.001
10	Donglvhua	4	4	1.000	0.096	0.225
11	Fuchi	2	2	1.000	0.058	0.070
12	Gouqi	15	2	0.133	0.003	0.020
13	Huaniao	13	13	1.000	0.968	0.784
14	Jinjishan	6	2	0.333	0.009	0.017
15	Jintang	21	11	0.781	0.020	0.168
16	Lidiao	6	1	—	0.000	0.001
17	Lujiazhi	7	2	0.286	0.007	0.020
18	Mayi	7	1	—	0.000	0.001
19	Mazhi	13	1	—	0.000	0.001
20	Miaozihu	7	1	—	0.000	0.017
21	Panzhi	13	4	0.423	0.004	0.025
22	Putuoshan	18	3	0.307	0.006	0.221
23	Qushan	21	10	0.733	0.023	0.001
24	Shengshan	13	5	0.538	0.034	0.092
25	Xiaochangtu	19	4	0.298	0.013	0.255
26	Xilvhua	4	3	0.833	0.072	0.193
27	Xiushan	19	4	0.298	0.038	0.363
28	Zhoushan	32	15	0.724	0.033	0.419

*Note:* When only a single haplotype of *T. muralis* is present on an island (*H* = 1), haplotype diversity (*H*_d_) is undefined and nucleotide diversity (*P*_i_) is calculated as 0.

**FIGURE 2:**
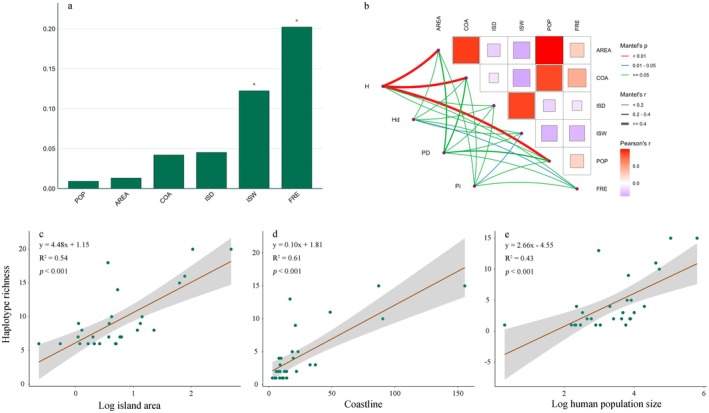
Multivariate analyses of genetic diversity driver. (a) Hierarchical partitioning reveals the independent effects of island characteristics on haplotype composition and distribution.*: *P* < 0.05. (b) Mantel tests assessing correlations between island characteristics and four genetic diversity metrics: Haplotype richness (H), Haplotype diversity (Hd), Phylogenetic diversity (PD), and Nucleotide polymorphism (Pi). (c–e) General linear models (GLMs) relating haplotype richness to: (c) log10 transformed island area, (d) coastline length, and (e) log_10_ transformed human population size. AREA, island area (km^2^); COA, coastline length (km); FRE, transportation frequency lever; ISD, distance to nearest island (km); ISW, land‐to‐water area ratio; POP, human population size.

The constrained ordination model, including all six environmental predictors, was statistically significant (*F* = 4.439, df = 6, 21, *p* = 0.002), explaining 43.3% of the total variation in haplotype distribution. Hierarchical partitioning of this explained variance revealed that the ISW and human transportation frequency lever (FRE) were the key drivers and the only predictors making significant independent contributions (ISW: independent contribution = 12.2%, *p* < 0.05; FRE: independent contribution = 20.20%, *p* < 0.05; Figure 2a). Among the 4 genetic diversity metrics examined, haplotype richness (H) exhibited significant associations with island area (AREA, Figure 2c), COA (Figure 2d), and human population size (POP, Figure 2e). In contrast, haplotype diversity (Hd) and nucleotide polymorphism (Pi) were primarily influenced by transportation frequency lever (FRE) and ISW, respectively (Figure 2b).

**FIGURE 3:**
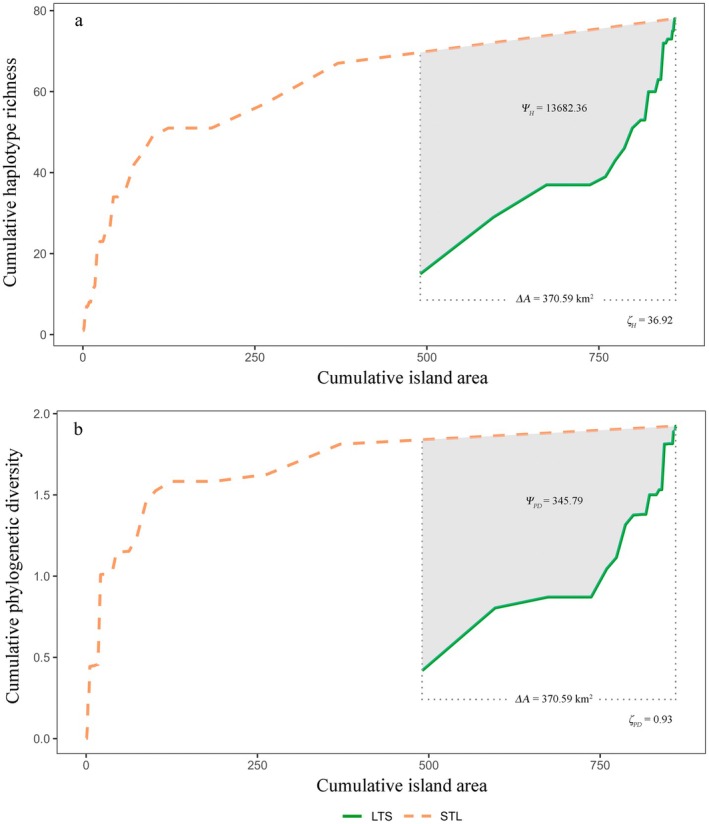
Diversity accumulation curves comparing LTS versus STL. (a) Haplotype richness, Δ*A* = 379.59 km^2^, *Ψ*_*H*_ = 13682.36, *ζ*_*H*_ = 36.92. (b) Phylogenetic diversity, Δ*A* = 379.59 km^2^, *Ψ*_*H*_ = 345.79, *ζ*_*PD*_ = 0.92.

The accumulation curves for both haplotype richness and phylogenetic diversity in *T. muralis* consistently demonstrated higher trajectories under the STL configuration compared to LTS (Figure 3). This pattern demonstrates that for any given total area, multiple small islands conserve greater haplotype richness and phylogenetic diversity than a single large island. To quantify this difference, we applied the ζ metric proposed by Mac Nally and Lake (1999), which yielded positive values for both measures (*ζ*_*H*_ = 36.92; *ζ*_*PD*_ = 0.93). Specifically, the STL approach conserved an additional 36.92 haplotypes per unit area relative to LTS, while phylogenetic diversity under STL exceeded LTS by 0.93 units per equivalent spatial extent.

## 4 Discussion

Our study provides direct support for the hypothesis that, under a fixed total area, an archipelago of several small (SS) islands conserves greater intraspecific genetic diversity in *T. muralis* than a single large (SL) island of equivalent area. This conclusion is evidenced by the consistently higher accumulation curves under the STL configuration and positive *ζ* values for both haplotype richness and phylogenetic diversity. Furthermore, we identified that both natural (land‐to‐water area ratio) and anthropogenic (transportation frequency) factors are key drivers shaping the spatial distribution of this genetic variation.

Phylogenetic diversity is commonly used to represent the total amount of biodiversity and to calculate the increase or decrease in diversity within a specific area (Faith 1992). Haplotypes can retain the integrity of a segment of genetic information and can be used to represent a specific type (Garg 2021; Zhang et al. 2024). Our study demonstrates that phylogenetic diversity indices can also account for the genetic variation of a species across locations within the same geographic region.

The distribution of ITS2‐based haplotypes is influenced by the water‐to‐land area ratio surrounding islands, and multiple genetic diversity indices exhibit varying degrees of correlation with island area. Larger islands typically possess greater resource availability, supporting larger population sizes and higher genetic diversity (Wang et al. 2012; White and Searle 2007). In the Zhoushan Archipelago, larger islands such as Zhoushan, Daishan, and Jintang support the majority of the human population. This anthropogenic activity has generated extensive concrete infrastructure—including walls, pavements, and bridges—which not only fulfills substrate requirements for *T. muralis* colonization but also creates heterogeneous microhabitat conditions through varied surface topography and moisture retention properties.

Human activities play a pivotal role in shaping the genetic diversity distribution patterns of wall‐dwelling bryophytes. As documented, anthropogenic construction of concrete structures such as walls and pavements provides *T. muralis* with novel, extensively distributed habitats that function as ecological analogs to natural rock substrates. These anthropogenic substrates frequently exhibit distinct physicochemical properties compared to natural rock formations (Francis 2011), potentially driving adaptive responses in moss populations. Furthermore, human‐mediated vectors significantly facilitate long‐distance spore dispersal. Transportation vectors (e.g., ships, vehicles) and human clothing can effectively transport spores across natural marine barriers, enabling colonization of distant islands (Yang et al. 2024).

Reproductive strategies are considered the primary factors influencing the levels of genetic variation and differentiation within bryophyte populations (Cronberg 2000). Despite the frequent occurrence of sexual reproduction and sporophyte production in *T. muralis* , molecular evidence indicates that its reproductive system is predominantly hermaphroditic (Longton 2006; Werner and Guerra 2004). Plants with predominantly self‐fertilizing systems often allocate a higher proportion of genetic diversity among populations while maintaining a smaller proportion within populations (Huang et al. 2021; Reutemann et al. 2024). However, this pattern may be reshaped by efficient spore dispersal mechanisms. Bryophyte spores possess strong long‐distance dispersal capabilities, and multiple small islands in different locations are more likely to receive spores from varied geographical sources. Large islands may maintain dominant haplotypes through clonal expansion, while small islands, due to resource limitations, are more likely to undergo repeated colonization‐extinction cycles, thereby preserving a greater number of rare haplotypes with short‐term persistence. In our study, the proportion of unique haplotypes on small islands reached 73.08%, whereas the most abundant haplotype on the largest island accounted for 75.41% of the specimens.

Indices such as haplotype diversity and nucleotide diversity have also been widely used in previous studies to assess intraspecific genetic variation (Caballero and García‐Dorado 2013; Fan et al. 2024). Haplotype diversity may serve as a more reliable indicator for evaluating the potential impacts of area changes on species populations (Fan et al. 2024). Nucleotide diversity is concerned with the long‐term evolutionary potential of populations, and large, continuous populations can maintain high levels of nucleotide diversity, which is crucial for adaptation in the context of current rapid global changes (Ord et al. 2023; Wang et al. 2019). The present study focuses solely on the cumulative calculation of haplotype number and phylogenetic diversity, lacking research on the evenness of haplotype distribution and long‐term evolutionary potential. Therefore, if the issue of cumulative calculation for these population genetic diversity indices can be resolved, it will be possible to investigate the genetic diversity of *T. muralis* in the Zhoushan Archipelago at different genetic levels. Synthesizing the results from different indices can provide a more comprehensive perspective and strategies for the design of nature reserves.

The ecological characteristics of natural islands differ markedly from those of habitat islands resulting from human activities. Natural island systems typically possess a long evolutionary history, characterized by relatively stable species composition (MacArthur and Wilson 1967). Habitat islands formed by human activities can generally be divided into two categories: original habitat islands and new habitat islands. Original habitat islands refer to island‐like patches formed by the gradual fragmentation and division of previously continuous habitats, such as forest fragments remaining after deforestation. Within these islands, as patch area decreases, habitats shrink and become isolated, genetic diversity tends to decline, and species gradually face the risk of local extinction (Fahrig 2003). In contrast, new habitat islands are “alternative habitats” created by novel environmental conditions within a matrix of non‐original habitats, such as islands in reservoirs and urban green patches, whose environmental characteristics may differ significantly from the surrounding matrix. These islands may exhibit relatively high diversity during the initial colonization phase, but over time, species/haplotypes that are poorly adapted to the local environment are gradually eliminated, leading to a stabilization or decline in diversity (Otálora et al. 2011). The linear relationship between island area and genetic diversity observed in our study is likely more applicable to original habitat islands. For new habitat islands, the genetic diversity of organisms may depend more on habitat quality than on area (Otálora et al. 2011). Therefore, when applying the SLOSS strategy, it is necessary to assess the history, connectivity, and environmental heterogeneity of habitat patches in specific contexts to inform the design of nature reserves.

Our findings should be interpreted in light of several study limitations. Methodologically, our application of the SLOSS framework was restricted to two cumulative diversity metrics—haplotype richness and phylogenetic diversity. This constraint, driven by data structure and the analytical challenges of comparing evenness or nucleotide diversity across fragmented configurations, implies that our support for “SS” strategry applies primarily to accumulated diversity. Empirically, while we identified significant environmental drivers, our models likely exclude unmeasured variables that influence genetic patterns, such as microscale substrate heterogeneity, precise spore dispersal vectors, or historical population bottlenecks. Finally, regarding generalizability, our results are rooted in a specific model system: a highly dispersible, selfing‐tolerant bryophyte on anthropogenic substrates. Caution is required when extrapolating the “SS” advantage to species with contrasting life history traits (e.g., dispersal‐limited or obligate outcrossing species) or to natural habitat islands. Rather than invalidating our results, these limitations define the scope of our study and highlight avenues for future research.

In conclusion, our study extends the SLOSS debate by demonstrating that, for intraspecific genetic diversity in *T. muralis* , a network of several small islands surpasses a single large island of equivalent area. This finding underscores the importance of considering both landscape configuration and anthropogenic factors in conservation planning. While the direct conservation urgency for this cosmopolitan species is limited, the methodological framework we developed—quantifying and comparing genetic diversity accumulation under different reserve configurations—provides a directly applicable tool for prioritizing habitats for endangered species in fragmented landscapes. Future work applying this framework to taxa of higher conservation concern will be crucial for validating its utility in optimizing real‐world reserve design.

## Funding

This work was supported by the National Natural Science Foundation of China, 32071643, 31570208. Natural Science Foundation of Shanghai, 21ZR1447400.

## Conflicts of Interest

The authors declare no conflicts of interest.

## Supporting information


**Data S1:** eva70229‐sup‐0001‐Supinfo.rar.

## Data Availability

All data associated with this study are provided in the Supporting Information.
